# Deep learning-based prediction of mandibular growth trend in children with anterior crossbite using cephalometric radiographs

**DOI:** 10.1186/s12903-023-02734-4

**Published:** 2023-01-17

**Authors:** Jia-Nan Zhang, Hai-Ping Lu, Jia Hou, Qiong Wang, Feng-Yang Yu, Chong Zhong, Cheng-Yi Huang, Si Chen

**Affiliations:** 1 Center of Orthodontics, Department of Dentistry, Sir Run Run Shaw Hospital, Zhejiang University School of Medicine, 3# Qingchundong Road, Hangzhou, China; 2 Department of Orthodontics, College of Stomatology, Zhejiang Chinese Medical University, 548# Binwen Road, Hangzhou, China; 3 School of Automation, Lishui Institute, Hangzhou Dianzi University, 1158# 2nd Street, Hangzhou, China; 4 Center of Orthodontics, Perfect Dental Care, 108# Xintang Road, Hangzhou, China; 5 Department of Orthodontics, Peking University School and Hospital of Stomatology, 22# Zhongguancun S. Ave., Beijing, China

**Keywords:** Deep learning, Mandibular growth, Prediction, Anterior crossbite, Convolutional neural networks

## Abstract

**Background:**

It is difficult for orthodontists to accurately predict the growth trend of the mandible in children with anterior crossbite. This study aims to develop a deep learning model to automatically predict the mandibular growth result into normal or overdeveloped using cephalometric radiographs.

**Methods:**

A deep convolutional neural network (CNN) model was constructed based on the algorithm ResNet50 and trained on the basis of 256 cephalometric radiographs. The prediction behavior of the model was tested on 40 cephalograms and visualized by equipped with Grad-CAM. The prediction performance of the CNN model was compared with that of three junior orthodontists.

**Results:**

The deep-learning model showed a good prediction accuracy about 85%, much higher when compared with the 54.2% of the junior orthodontists. The sensitivity and specificity of the model was 0.95 and 0.75 respectively, higher than that of the junior orthodontists (0.62 and 0.47 respectively). The area under the curve value of the deep-learning model was 0.9775. Visual inspection showed that the model mainly focused on the characteristics of special regions including chin, lower edge of the mandible, incisor teeth, airway and condyle to conduct the prediction.

**Conclusions:**

The deep-learning CNN model could predict the growth trend of the mandible in anterior crossbite children with relatively high accuracy using cephalometric images. The deep learning model made the prediction decision mainly by identifying the characteristics of the regions of chin, lower edge of the mandible, incisor teeth area, airway and condyle in cephalometric images.

## Background

Class III malocclusion is a frequently observed clinical problem, mostly manifested as anterior crossbite, occurring in 4–14% East Asian populations [1]. Anterior crossbite not only affects the occlusal function, but also damages the balance of profile, and increases the social and psychological burden of the child [2]. As for the components of Class III malocclusion, the combination of underdeveloped maxilla and overdeveloped mandible was most common at 1/3, whereas those with a normal maxilla and overdeveloped mandible constituted about 1/5 [3]. Children who have Class III malocclusion because of excessive growth of the mandible are extremely difficult to treat. Prediction for the future growth trend of the mandible greatly affects the decision for treatment option. Inaccurate prediction may lead to insufficient or unnecessary treatment.

In fact, it is difficult for orthodontists to accurately predict the remaining growth amount of the mandible. Similar anterior crossbite in the mixed dentition may develop to malocclusions with different skeletal pattern in the permanent dentition [4]. Therefore, accurate prediction of the future mandibular growth in the early stage will be very useful to assist treatment planning and prognosis.

Various mathematical methods have been proposed over the past few decades to predict the growth of mandible. Sato et al. [5] established a linear equation to predict mandibular growth potential based on bone age, which was assessed from hand-wrist radiographs. This method was not widely used due to the unnecessary radiation dose to children resulted from the additional hand-wrist radiograph. Later, Mito et al. [6] developed a formula to predict mandibular growth potential by calculating the actual growth of the mandible (condyle-gnathion) with cervical vertebral bone age, which was evaluated from cephalometric radiographs. However, this method was not suitable for individual prediction because the formula was derived from the mixed data of 7–13 years of age children, including different stages of the growth period. Moshfeghi et al. [7] set a regression equation to predict mandibular length (Articulare-Pogonion) by analyzing the morphological changes of the cervical vertebrae on lateral cephalograms. Recently, Franchi et al. [8] developed a mathematical mixed effect model to predict the growth of mandible, and the results demonstrated that cervical stage, chronological age and gender were significant predictors for the annualized increments in mandibular growth.

Among the above studies, regression equation analysis is the most frequently used method for predicting mandibular growth potential. Regression equation is applied to identify and verify the factors that probably reflect the growth potential of mandible [9]. However, as it works based on the analysis of a linear combination of covariates, it may be too simplistic to predict the complex growth outcome of mandible.

Deep learning technique is a big breakthrough in machine learning field, and it has showed great application potential in medical filed in recent years. Deep learning models have behaved high accuracy on medical image classification by automatically learning from datasets, which are manually annotated by clinical experts [10, 11]. Among deep learning models, the convolutional neural network (CNN) achieves the most attention and is widely researched due to its amazing performance for detection of medical images [12]. CNN models can automatically learn and extract characteristic features and structures from training images, and then make classification and prediction for new images.

In the past few years, deep learning techniques have been successfully applied to the dental field and acquired significant achievements. Deep learning models have showed a reliable ability in identifying and classifying several types of dental imaging, even surpassing human experts [13, 14]. Kim et al. [15] used a deep CNN model to automatically identify and classify skeletal malocclusions into three classes from 3D Cone-Beam Computerized Tomography (CBCT) craniofacial images and achieved a good performance of the accuracy over 93%. Yu et al. [16] constructed a multimodal CNN model to provide vertical and sagittal skeletal diagnosis with cephalometric radiographs and reported a high classification accuracy at 96.4%. The CNN model has showed its great potential for the analysis of Orthodontic X-ray images, including CBCT and cephalograms. However, to our knowledge, prediction for the growth potential of the mandible based on CNN models has not yet been reported.

In this research, we aimed to train a deep learning CNN model to automatically classify the mandibular growth result for children with anterior crossbite into two types- normal and overdeveloped, using cephalometric radiographs. We also aimed to reveal the critical regions in cephalometric images based on which the model made the classification.

## Methods

### Patients inclusion

This retrospective study was approved by the Research Ethics Committee of Sir Run Run Shaw Hospital, Zhejiang University School of Medicine (No. 20210729-122). In this study, 512 patients who visited our Orthodontic Center between January 2010 and December 2016 with the chief complain of anterior crossbite were screened for further research.

The inclusion criteria were as follows: (1) anterior crossbite; (2) Class III or Class I molar relationship; (3) ANB < 0°; (4) without functional mandibular setback to edge to edge; (5) 8–14 years of age; (6) availability of the pre-treatment (T1) and after 18-year of age (T2) lateral cephalograms which were of good quality.

The exclusion criteria were as follows: (1) maxillary retrusion; (2) anterior crossbite caused by misaligned teeth; (3) congenital deformity such as cleft lip and palate, infection or trauma history.

A total of 296 patients were included in this study (142 males and 154 females, ranged from 8.08 to 13.92 years, with an average age of 10.8 years).

### Cephalometric analysis and skeletal classification

All T1 and T2 cephalometric radiographs were uploaded into Dolphin software (Version 11.9, Dolphin Digital Imaging, Chatsworth, Calif, USA). The anatomic contours were traced and cephalometric landmarks were located simultaneously by two orthodontic experts. Any disagreements about landmark location were resolved by retracing the anatomic contours until the two experts achieved the same point.

The cephalometric measurements related to evaluate the growth condition of mandible included SNB, ANB, Wits appraisal, FMA (mandibular plane to FH), SNPog (facial plane to SN), NSGn (Y-Axis), NSAr (Sella Angle), ArGoMe (Gonial Angle); Ar-Gn (effective mandibular length), Co-Gn (total mandibular length), Go-Gn (mandibular body length), and Co-Go (mandibular ramus length).

Anterior crossbite with prognathic mandible belongs to skeletal malocclusion which may require orthognathic surgery according to the Kerr’s research [17]. In the contrast, anterior crossbite with normal mandible can be treated by orthodontics. According to the cephalometric analysis results at T2, if SNB > 86°, ANB < − 2° and Wits value < − 2.0 mm [17], the subject was recognized as a patient with overdeveloped mandible and assigned to Group A, otherwise, assigned to Group B (patient with normal mandible). Finally, 102 patients (49 males and 53 females, ranged from 8.08 to 13.92 years, with an average age of 11.5 years) were sorted to Group A and 194 patients (93 males and 101 females, ranged from 8.08 to 13.83 years, with an average age of 10.4 years) were sorted to Group B.

### Datasets build and annotation

The lateral cephalometric images of the 296 subjects at T1 were collected for the training and testing of the deep learning-based CNN model. Among those, 256 lateral cephalograms (82 images from Group A and 174 images from Group B) were randomly selected as the training dataset. During the process of model training, the tenfold cross-validation process was applied to acquire the best model parameters. The remaining 40 cephalometric images (20 images from Group A and 20 images from Group B) were used as the testing dataset to evaluate the performance of the deep learning-based CNN model.

Area adjustment of the cephalometric images was conducted in the process of preparing square images for the deep learning model generation. As the redundant information in the cephalometric images would affect the efficiency of the training process, the input images were zoomed out, cropped and resized to 512 * 512 pixels without changing its aspect ratio. The specific requirements of reducing the region were as follows: the right border of the image should include the complete nose structure and the least air area; the bottom border of the image should include the complete chin structure and the least air area; the top border of the image should include the complete temporomandibular joint structure and part of skull base structure; the left border of the image should include the complete upper airway structure and part of cervical vertebra structure.

Then, in order to avoid overfitting, the images were randomly augmented by applying random transformations, including rotation, horizontal and vertical flipping, width and height shifting, shearing, and zooming.

Based on the classification results of Group A and Group B, the reference annotations of the mandibular growth trend (overdeveloped vs normal) for T1 cephalograms were created.

### Architecture of the deep-learning model

**Fig. 1:**
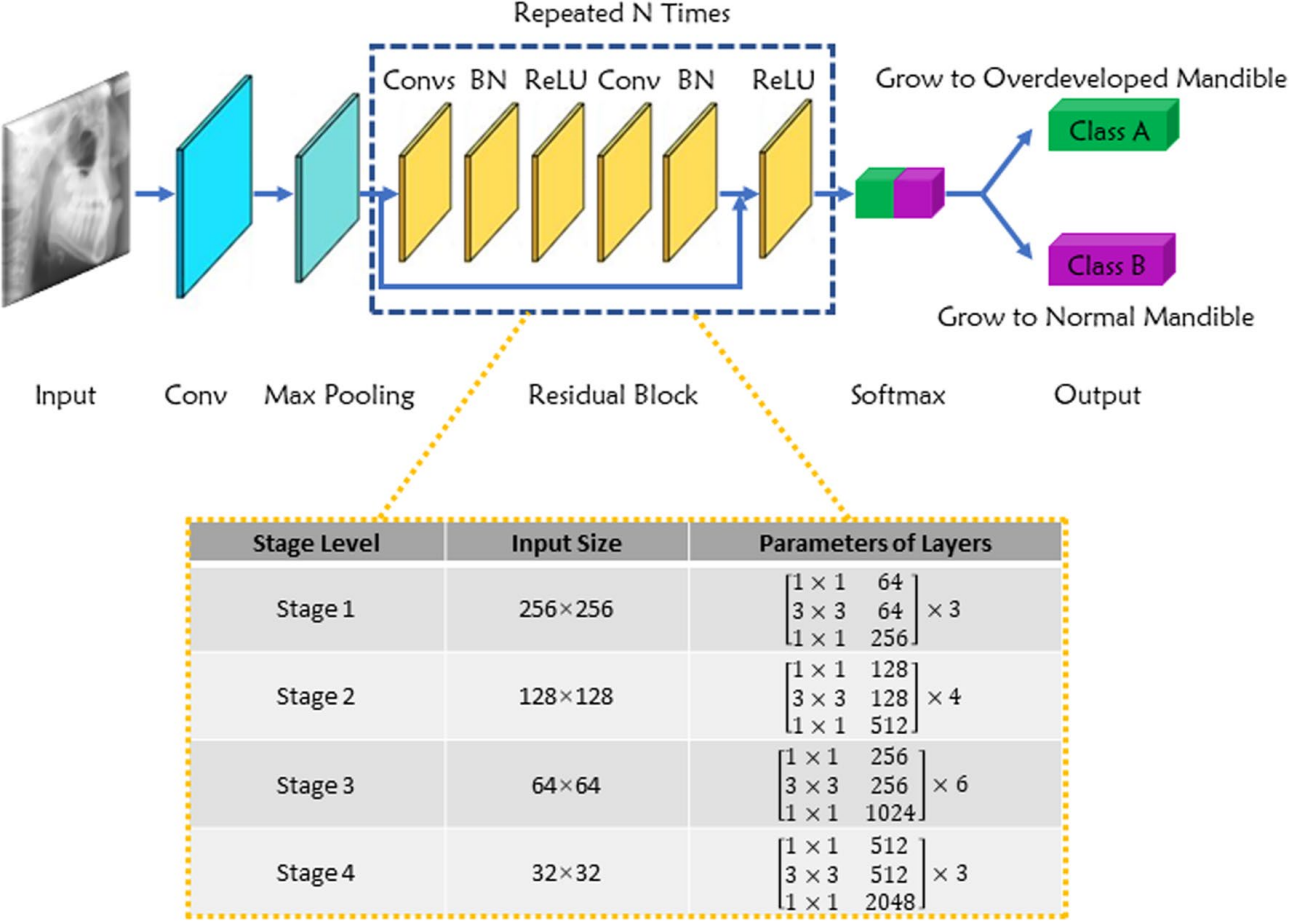
The work flow and architecture of the deep learning model. *Conv* convolution, *BN* batch norm, *ReLU* rectified linear unit

We developed a neural network based on ResNet50, which was famous for its excellent performance in image classification and object detection [18]. The architecture of this model was composed of several residual networks and a softmax layer. The residual network was used to detect and analyze the characteristics of the input images. The softmax layer was adopted to predict the classification of the object. Figure 1 showed the workflow of this deep learning-based CNN model. The training process was performed on a Linux machine with a GPU accelerator, and the initial learning rate and training epoch was 0.01 and 300, respectively.

### Visualization of region of interest

The analysis behavior and process of the model was recorded and visualized by marking a region of interest (ROI) on the input image, using a visualization method called “Grad-CAM”. Grad-CAM is a region proposal network that is equipped into the output layer of the neural network and can mark the ROI [19]. Specifically, Grad-CAM has the super advantage of localizing the most discriminative and critical region from the whole scene for classifying the input image because some special spatial element in the feature maps plays an essential role in the calculating and prediction process of the model.

### Mandibular growth prediction and statistical analysis

**Fig. 2:**
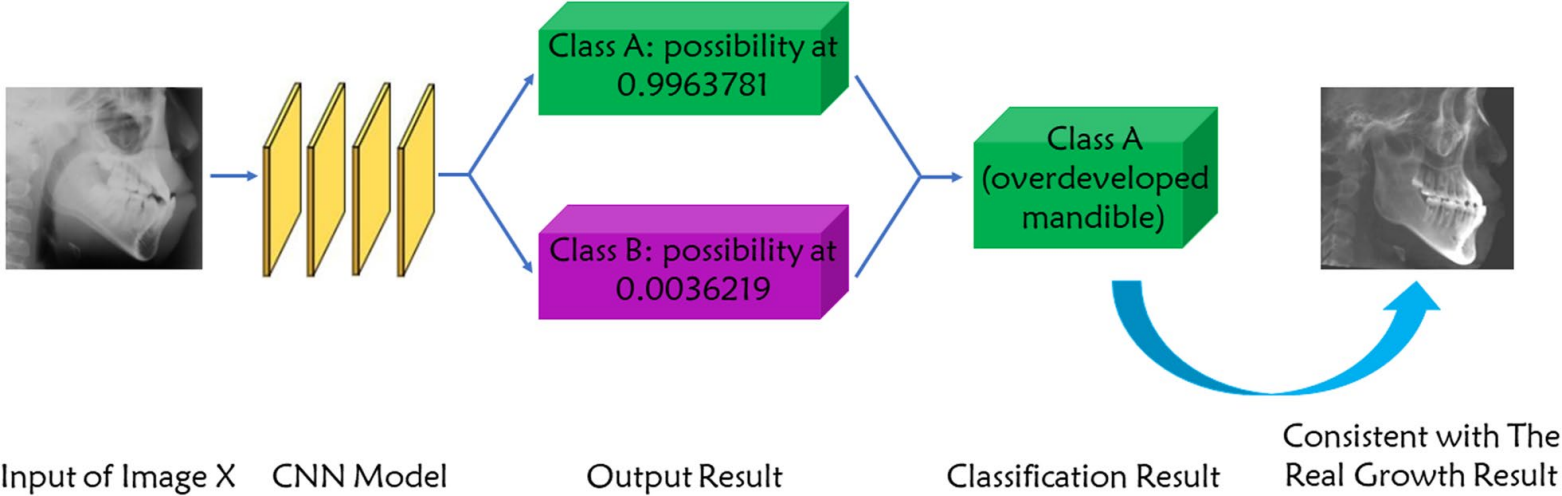
The example of the classification process by the deep-learning CNN model. Class A: grow to overdeveloped mandible. Class B: grow to normal mandible. The classification result was verified by the real growth result according to the lateral cephalogram after 18-year of age

After completion of the training process of the CNN model, the testing dataset (20 images from Group A and 20 images from Group B) was classified by the CNN model and junior orthodontists respectively. For CNN model classification, the testing images were input to the model and the classification result will be given based on the possibility comparison between different classification. For example, as shown in Fig. 2, after the input of Image X, the output result was shown as: 0.9963781 for Class A (overdeveloped mandible) and 0.0036219 for Class B (normal mandible). Then Image X was classified to Class A. For junior orthodontist classification, three junior orthodontists (clinical work experience less than 5 years) gave their individual prediction (only one prediction result: Class A or Class B) for the future mandibular growth of the subject based on cephalometric measurements and analysis of the testing cephalogram.

The prediction results from the deep learning-based CNN model were compared with those from the junior orthodontists to explore its clinical application by the following indices: classification accuracy, true positive rate (sensitivity), false negative rate, false positive rate, true negative rate (specificity), and the area under the curve (AUC) [20]. These calculating work were based on a Keras framework in Python.

## Results

### Comparison of prediction results

**Fig. 3:**
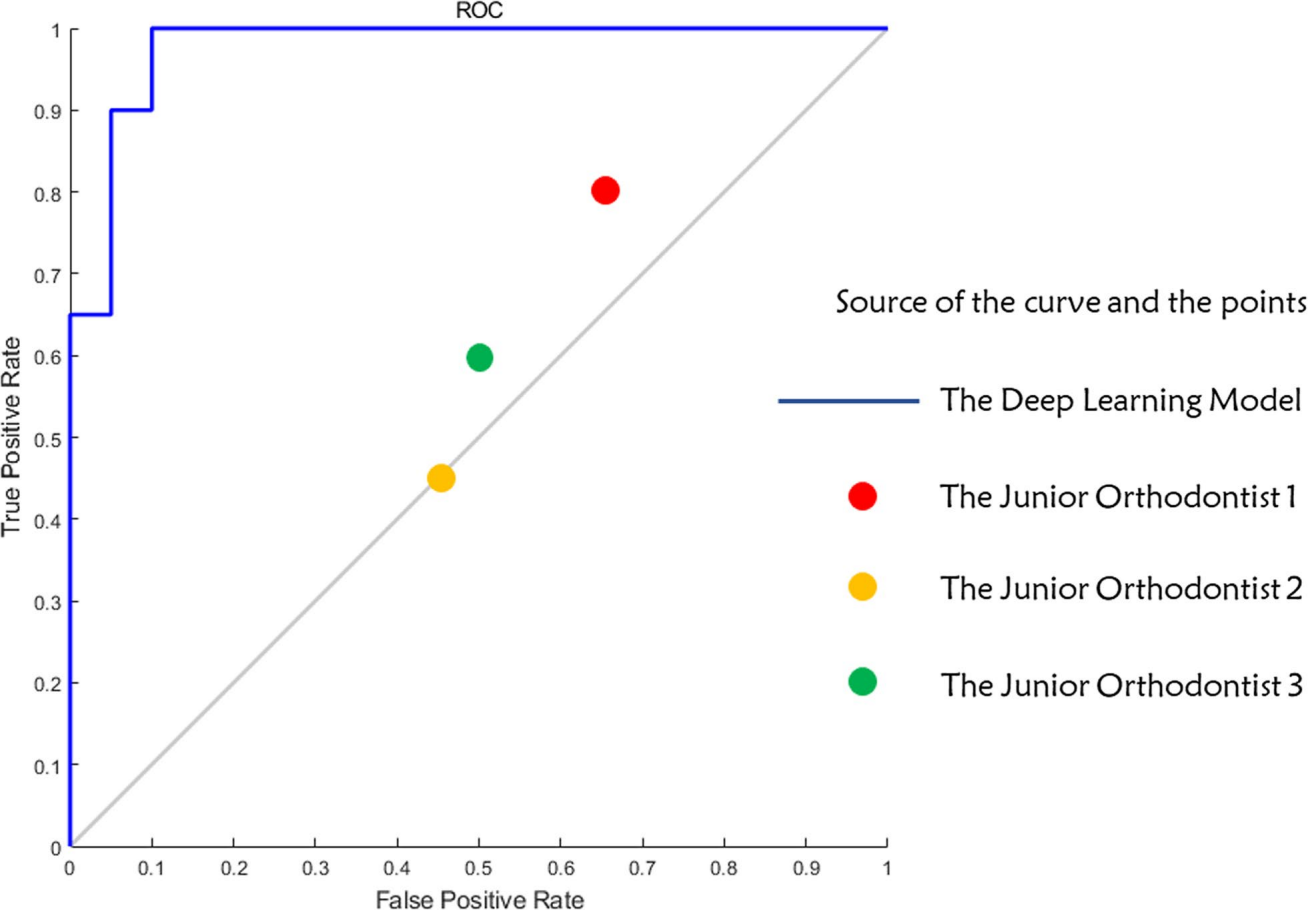
Receiver operating characteristic (ROC) curve of the deep learning model and the performance points of the junior orthodontists. The solid bule line displayed the trajectory of the deep learning model with respect to Sensitivity (True Positive Rate) and 1-Specificity (False Positive Rate). The colorful points represented the performance of the junior orthodontists. None of the junior orthodontists outperformed the deep learning model

The performance of the deep-learning CNN model and the junior orthodontists were summarized in Table 1. Higher accuracy was found in the deep-learning model prediction results (85.0%) when compared with that of the three junior orthodontists (54.2%). The mean sensitivity/specificity for the model prediction and human prediction were 0.95/0.75 and 0.62/0.47 respectively. The receiver-operating characteristics (ROC) curve of the model and the performance points of the junior orthodontists in the ROC picture were shown in Fig. 3. The AUC value of the deep-learning model was 0.9775.

**Table 1:** Performance of the deep learning model and the junior orthodontists

	Accuracy (%)	TPR	FNR	FPR	TNR	AUC
The deep learning model	85	0.95	0.05	0.25	0.75	0.9775
The junior orthodontists (mean value)	54.2	0.62	0.38	0.53	0.47	/
Orthodontist 1	57.5	0.8	0.2	0.65	0.35	/
Orthodontist 2	50	0.45	0.55	0.45	0.55	/
Orthodontist 3	55	0.6	0.4	0.5	0.5	/

*TPR* true positive rate (sensitivity), *FNR* false negative rate, *FPR* false positive rate, *TNR* true negative rate (specificity), *AUC* area under the curve

### Visualization of localization results

**Fig. 4:**
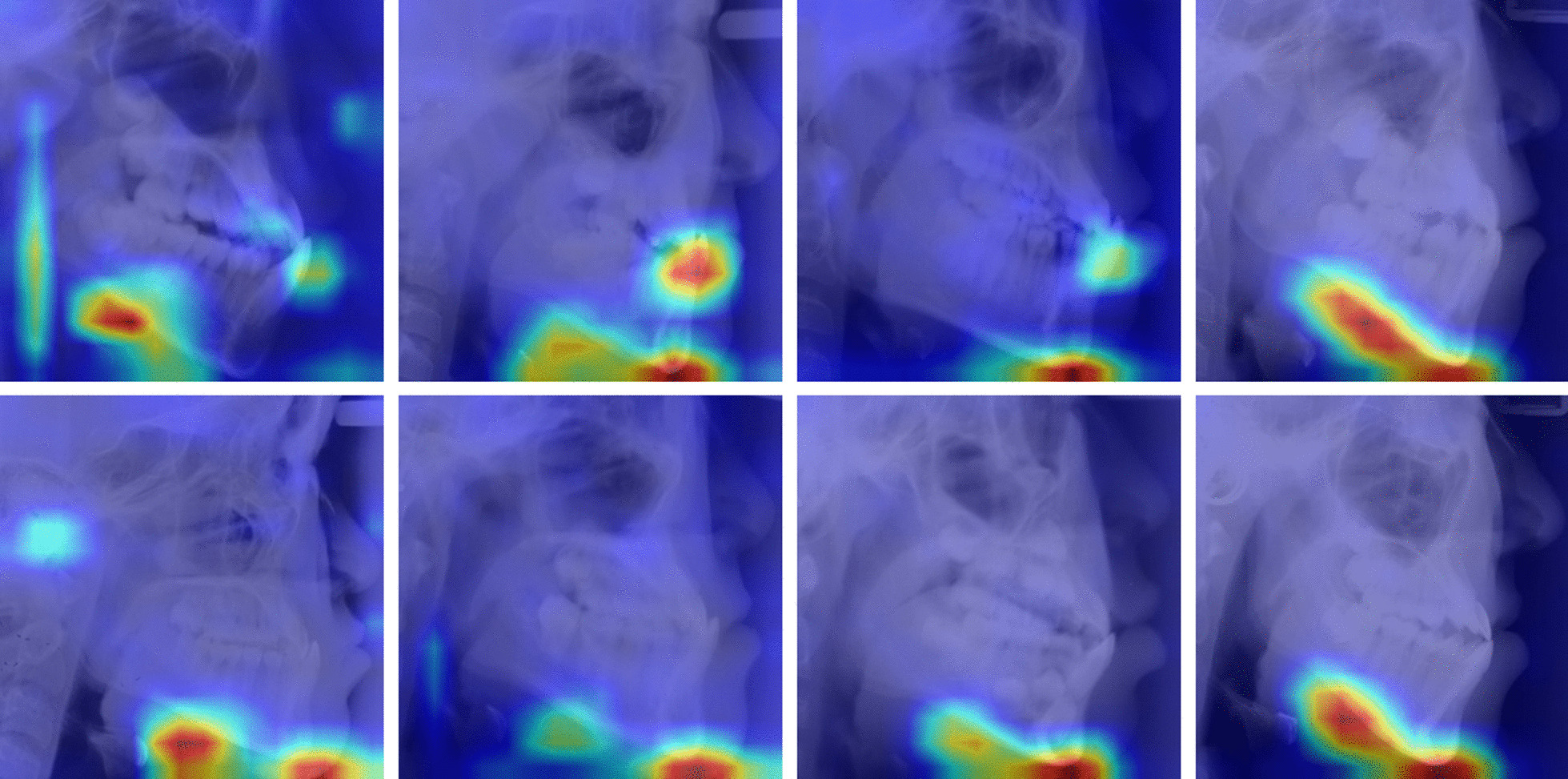
Examples of Grad-CAM localizing ROIs in the cephalometric radiographs. Red colors indicated areas of high influence on the classification making, whereas bule colors indicated areas of low influence

Figure 4 showed some examples of the heatmap images reflecting the characteristic of the learning and classification behaviors of the deep-learning model. Visual inspection results showed that the deep-learning model mainly focused on the following areas: chin (40/40), lower edge of the mandible (28/40) and incisor teeth (7/40). It’s interesting to find that the area used to be considered as not important for the prediction of the mandibular growth such as the airway area was recognized as ROI in some subjects by the deep-learning model (2/40) (Table 2).

**Table 2:** The occurrence times of the regions of interest among the heatmaps of the 40 testing images

	Chin	Lower edge of the mandible	Incisor teeth	Airway	Condyle
The occurrence times	40	28	7	2	2

## Discussion

Class III malocclusion can be of dental or skeletal origin, so it is crucial to classify the malocclusion accurately in order to manage it on a sound clinical basis. Class III malocclusion resulted from overdeveloped mandible is extremely difficult to treat. The mandible has the longest growth and develop period among the craniofacial bones [21]. Therefore, the prediction for the mandibular growth in the early stage is very challenging. Severe skeletal Class III with prognathic mandible requires orthognathic surgery. However, if camouflaged orthodontic extraction treatment was conducted at an early stage, the result might be unstable or even deteriorated during the treatment.

Since the key point of successful treatment of anterior crossbite lies on the mandible, the early and accurate prediction of the growth trend of mandible and treatment with corresponding interventional methods can be beneficial to the patients [22]. Clinically, the decision of proper treatment and timing for Class III patients mainly relies on the clinical experience of orthodontists. However, individual clinical experience varies significantly among different orthodontists. On the other hand, even for experienced orthodontists, it is also challenging to accurately predict the remaining growth of mandible when facing anterior crossbite in mixed dentition or early permanent dentition.

In this study, a deep learning-based CNN model was trained to predict the growth trend of mandible in anterior crossbite child from pre-treatment cephalometric radiographs. The result showed that the deep learning model achieved 85% accuracy in predicting whether the mandible of anterior crossbite child will grow into an overdeveloped mandible or a normal mandible. The accuracy of the machine learning based prediction is much higher than that from the junior orthodontists, which was 54.2%. The sensitivity and specificity for the model prediction were also higher when compared with human prediction (0.95 vs 0.62 and 0.75 vs 0.47). The reason for the good performance of this deep learning model should be related to the innovation of the prediction process, which replaced the analysis of linear and angular measurements by a direct and comprehensive detecting and analyzing system [23]. This deep learning model was developed from a well-known algorithm called ResNet, which has been recognized as a breakthrough innovation for its strong ability to train extremely deep neural networks, possessing important advantages such as analyzing more structure information, relevance information and detail information of images [24]. In the deep learning field, the AUC value is considered to be an important index when evaluating the performance of the models [25]. The deep learning model in this study achieved an AUC value of 0.9775, demonstrating its reliable performance.

Among the results from this study, there were two that worth more notice. One was that the false positive rate of the deep learning model was much lower than that of the junior orthodontists. This suggested that orthodontists with less clinical experience tended to be overcautious in the prediction of future mandibular growth in order to reducing clinical risk and avoiding medical dispute. Another revealing was that the false negative rate of the deep learning model was also much lower than that of the junior orthodontists. This implied that junior orthodontists might be more likely to make a wrong prediction of judging the mandible to be normal in the future, whereas the mandible would grow into an excessive size. This type of wrong prediction may result in unsuccessful camouflaged orthodontic treatment for cases which actually need orthognathic surgery and anterior crossbite relapse after treatment.

In addition, we combined Grad-CAM into the deep learning model to provide a guide for visually predicting the growth feature of the mandible in anterior crossbite child and reveal the prediction mechanism behind. The output of this model includes heatmaps, which allowing the identification of the main areas in cephalometric images by which the model made the prediction decision. The results showed that the heatmaps included regions of chin, lower edge of the mandible, incisor teeth area, airway and condyle. The regions like chin and lower edge of the mandible are easy to understand as they are closely related to the growth regulation of mandible [26, 27]. However, the airway region is unexpectable as we rarely take it into consideration for mandibular growth prediction in anterior crossbite child. This interesting result could be further understood by some reports claiming that the morphological characteristics of the airway were correlated with the craniofacial skeletal pattern, such as skeletal Class II malocclusion (mainly manifested as mandibular retraction) [28] and skeletal Class III malocclusion (mainly manifested as mandibular protrusion) [29, 30]. The findings from this study provided some new clues for prediction.

Despite the good performance of the deep learning-based CNN model, there are several limitations in our approach. First, the total size of the training dataset was small, as well as the testing dataset. Second, other deep learning models were not applied to compare the prediction performance. Last, clinical characteristics and family history of patients were not included in the algorithm, so it is unknown whether the performance of the deep learning model could be improved if these factors were added.

## Conclusions

1. The deep learning model behaved well and resulted in a much higher accuracy in mandibular growth trend prediction for children with anterior crossbite.
2. The deep learning model made the prediction decision mainly by identifying the characteristics of the regions of chin, lower edge of the mandible, incisor teeth area, airway and condyle in cephalometric images.

## Data Availability

The datasets used and/or analyzed during the current study are available from the corresponding author on reasonable request.

## Abbreviations

CNN: Convolutional neural network
CBCT: Cone-beam computerized tomography
Conv: Convolution
BN: Batch norm
ReLU: Rectified linear unit
ROI: Region of interest
AUC: Area under the curve
TPR: True positive rate
FNR: False negative rate
FPR: False positive rate
TNR: True negative rate
ROC: Receiver-operating characteristics
